# Evaluation of pan-alimentary MRI examinations: intrasubject variability and examination duration

**DOI:** 10.1007/s00261-026-05399-z

**Published:** 2026-04-17

**Authors:** Fredrika Sofia Magnuson, Tina Okdahl, Davide Bertoli, Isabelle Myriam Larsen, Nina Marie Videbech, Maja Veitland Ilkjær, Jens Brøndum Frøkjær, Asbjørn Mohr Drewes, Klaus Krogh, Esben Bolvig Mark

**Affiliations:** 1 Department of Hepatology and Gastroenterology, Aarhus University Hospital, Aarhus, Denmark; 2 Department of Clinical Medicine, Graduate School of Health, Aarhus University, Aarhus, Denmark; 3 Mech-Sense, Department of Gastroenterology and Hepatology, Aalborg University Hospital, Aalborg, Denmark; 4 Thisted Research Unit, Aalborg University Hospital, Thisted, Denmark; 5 Department of Clinical Medicine, Aalborg University, Aalborg, Denmark; 6 Radiology Research Center, Department of Radiology, Aalborg University Hospital, Aalborg, Denmark

**Keywords:** Magnetic Resonance Imaging, Gastrointestinal Motility, Intrasubject Variability, Gastric Emptying, Small Bowel, Colon

## Abstract

**Background:**

Many gastrointestinal (GI) disorders entail disturbances in GI functions such as motility and volume. Magnetic resonance imaging (MRI) can assess GI motility and volumes in one single examination; however, challenges with clinical implementation with pan-alimentary MRI can be intrasubject variability and time-consuming examinations.

**Objectives:**

This study aims to examine 1) intrasubject variability in MRI-assessed gut volumes, motility and whole-gut transit time 2) propose a shortened pan-alimentary MRI examination, and 3) demonstrate feasibility of clinical implementation of pan-alimentary MRI.

**Methods:**

Five individual MRI studies were included in this study. The intrasubject variability of MRI measurements was assessed in 40 healthy subjects scanned twice with more than two weeks between examinations. Parameters were assessed with the intraclass correlation coefficient (ICC) and included gastric half-emptying time, small bowel motility index, whole gut transit capsule score, and segmental volumes of stomach, small bowel and colon. A shortened MRI examination was tested using estimations of gastric half-emptying time calculated using either a five datapoint- and a three datapoint postprandial MRI examination, performed in 86 individuals. Feasibility of clinical implementation was exemplified by two patient cases.

**Results:**

Gastric half-emptying time showed moderate reproducibility (ICC: 0.53 [95% CI: 0.31-0.74]). Fasting small bowel motility index had an ICC of 0.66 [0.45-0.82], and fasting total colon volume had an ICC of 0.47 [0.23-0.72]. Whole gut transit capsule scores had poor reproducibility (ICC: 0.27 [0.04-0.76]). Reducing scan datapoints for calculating gastric half-emptying time, maintained accuracy (concordance correlation coefficient: 0.93-0.95). Feasibility of implementation was shown by successful examinations at both a minor- and a university hospital.

**Conclusion:**

Despite fluctuating ICCs of intrasubject variation measurements, physiological variances are to be expected when assessing the GI tract. The proposed shortened MRI examination is applicable outside specialized research settings and may provide new insights in patients with motility disorders.

**Supplementary Information:**

The online version contains supplementary material available at https://doi.org/10.1007/s00261-026-05399-z.

## Introduction

Gastrointestinal (GI) motility refers to the complex coordinated muscle contractions that propel, process, and ultimately expel food, liquids, and gas through the digestive tract [1, 2]. Impairment in these functions, termed GI *dysmotility*, are often severely disabling and a common complication across a range of diseases such as diabetes mellitus, achalasia, multiple sclerosis, and Crohn’s disease, and often results in debilitating symptoms such as constipation, diarrhea, incontinence, pain, bloating, nausea, and early satiety. Symptoms are typically non-specific, correlate poorly with clinical findings and remain diagnostically challenging [2–4]. There is no golden standard for measurements of GI motility [4]. Current diagnostic modalities – such as endoscopy, esophageal/gastroduodenal/anal manometry, scintigraphy, and radiopaque marker transit measures, can be invasive, uncomfortable, require specialized knowledge, or lack standardization [4–6].

GI dysmotility frequently affects multiple GI segments; thus, a comprehensive, non-invasive and standardized modality assessing the dynamic physiology in all GI segments in a single examination is desirable. Magnetic resonance imaging (MRI) provides high spatial resolution, radiation-free and noninvasive assessment of the entire GI tract [7]. General challenges with the imaging of the GI tract can be artefacts due to the presence of air in the lumen, organs’ motility, and breathing. MRI is effective in detecting both motility and physiological abnormalities throughout the entire GI tract, all in one examination [8, 9]. Nonetheless, despite its potential, clinical implementation is limited by a lack of standardization, variability in post-processing and interpretation, restricted availability, and long acquisition and analysis time that increase costs [7]. Therefore, due to the rapidly growing development of MRI techniques, new, clinically relevant and effective protocols are required to clarify and facilitate clinical use. Based on pooled results from previous research trials, this study proposes one single pan-alimentary MRI examination, that includes individual measurements of GI parameters, is clinically relevant and logistically feasible [10–12]. This examination produces a highly detailed assessment of the volume, content, and motility of most parts of the GI tract in one MRI scan session lasting less than two hours. This study aims to: (1) Separately, evaluate day-to-day intrasubject reproducibility in MRI-assessed volumes of the stomach, small bowel, and colon, as well as gastric half-emptying time, small bowel motility, and whole gut transit capsule score; (2) propose a shortened MRI scan examination reducing time spent in the scanner, making clinical use and implementation, more accessible and applicable; (3) to demonstrate the feasibility of clinical implementation of the MRI examination, outside research settings in two other hospitals exemplified by two clinical cases.

## Materials and methods

### Data collection and ethical approval

Data were collected from five different studies conducted in Denmark, as shown in Table 1. Three studies were previously published clinical trials conducted at Aalborg University Hospital and approved by the North Denmark Region Committee on Health Research Ethics (N-20200102 and N-20190020) and Clinical Trials Information System EudraCT no. 2022-500108-23-00. [10–12] One study was an ongoing clinical trial performed at Aarhus University Hospital and approved by the Central Denmark Region Committee on Health Research Ethics (1-10-72-369-21). One study was a local quality assurance project performed at Thisted Hospital (ID: K2022-023). Written informed consent was obtained from all participants in the studies.

### MRI studies

The MRI studies had different endpoints as the primary purposes of the studies varied. Nonetheless, the MRI examinations generally included the same type of measurements: a pan-alimentary assessment of the different segments’ content, volume, and motility.

 Participants in all studies were scanned in the morning after a minimum of 6 h of fasting for food and two hours for water. Images of the stomach, small bowel, and colon were obtained before and after a test meal. An MRI protocol overview is shown in Table 1. Specific MRI scan parameters are shown in the supplementary material in Table S1.1, Table S1.2, and Table S1.3. This article included the following MRI-derived measures: Gastric half-emptying time, gastric content volume, small bowel volume, small bowel motility index, colon volume (total and segmental volumes), and whole gut transit marker capsules (position score).

**Table 1:** Overview of the five included MRI studies

Author and year	Population, *n*	Design	Intervention	Timepoints for repeated scans, min
Bertoli et al. 2023[12]	40 HV, 46 DM	Observational	Meal (300 kcal): 400 ml nutrient drink and water.	Fasting, 0, 15, 75, 90, 105
Mark et al. 2024[10]	20 HV	Cross-over	Meal (285 kcal): Three granola bars + 400 ml water.	Fasting, 0, 15, 45
Larsen et al. 2024[11]	20 HV	Cross-over	Meal (285 kcal): Three granola bars + 400 ml water.	Fasting, 0, 15, 45
Magnuson et al. unpublished	1 SCI	Case description	Meal (300 kcal): 400 ml nutrient drink and water.	Fasting, 0, 15, 30, 45, 60, 75
Mark et al. unpublished	1 FD	Case description	Meal (300 kcal): 400 ml nutrient drink and water.	Fasting, 0, 75

HV, healthy volunteers; DM, patients with diabetes mellitus; SCI, patient with spinal cord injury; FD, patient fulfilling ROME IV criteria for Functional Dyspepsia.

### MRI measurements

No single clinical sequence can assess both motility and structural parameters with sufficient details. The MRI measurements evaluated in this study are obtained in a single MRI examination using five independent MRI scan sequences that individually is used to quantify: (1) gastric volumes and half-emptying time, (2) small bowel volume and motility, (3) colon segmental volumes, and (4) whole gut transit marker capsules. The specific measurements include fasting and postprandial volumes of stomach and small intestine, and fasting volumes of total and segmental colon and the position of marker capsules.

#### Gastric content volume and half-emptying time

Images of the stomach were performed using static T2-weighted scans repeated at multiple timepoints. Gastric content volumes comprising both liquid/solid contents were manually segmented using a custom analysis framework (MATLAB v.R2019b, MathWorks, Natick, MA, USA). Regions exhibiting high signal intensity within the gastric lumen were interpreted as liquid content, whereas low-intensity areas located in the anterior portions of the stomach were classified as gas. The gas volume was not included in any calculations. The time point for half-emptying of the stomach’s content volume at the 0-min scan (T_50_) was calculated using an online analysis tool (apps.menne-biomed.de/gastempt/) [13]. All calculations of T_50_ were estimated in one batch calculation for each of the included studies.

#### Small bowel volume and motility

Images of the small bowel were performed using dynamic CINE scans that captures motility over time. Five slices of 20s dynamic coronal images were obtained. One slice was obtained per breath hold. Data were analyzed on the online platform Entrolytics (Motilent, Ford, UK). The small bowel was delineated using a brush tool, and the segmented volume represented the small bowel volume. The GIQuant^™^ (Motilent, Ford, UK) algorithm generated motility maps for each dynamical scan [14]. The small bowel motility index, GIQuant score, and the delineated small bowel volume were reported for each scan time point. The motility index is an arbitrary unit representing more motility with higher numbers and less motility with lower numbers; however, the direction of contractions is not obtained using this method.

#### Colon segmental volumes

Images of the colon were obtained using coronal T2-weighted images. These were used to analyze colonic total and segmental (ascending, transverse, descending, and sigmoid/rectum) volumes. The images were analyzed using a semi-automatic analysis framework in MATLAB (v.R2019b, MathWorks, Natick, MA, USA). [15] The segmented volumes included fecal content and gas.

#### Whole gut transit marker capsules

Images of the marker capsules were obtained with a coronal DIXON LAVA-Flex sequence with an acquisition time of 20 s during a single breath hold. Three MRI marker capsules were swallowed 24 h before the scheduled MRI scan [10, 16]. The capsules were 21.5 mm x 8.3 mm and consisted of two half-shells made of medical grade polyetheretherketone resin KETRON PEEK-1000 natural. The capsules were hand-filled for these particular research studies, with a solution of MRI contrast fluid (Gadoteridol 279.3 mg/ml, Prohance^®^, Bracco International B.V., Amsterdam, The Netherlands) and tap water mixed 1:10 to ensure high imaging contrast [17].

The capsules’ position in the GI tract was rated using the scale proposed by Chaddock et al [16]. The rating scale is as follows: Small bowel: 7, proximal ascending colon: 6, distal ascending colon: 5, proximal transverse colon: 4, distal transverse colon: 3; descending colon: 2, sigmoid colon and rectum: 1, expelled: 0. The mean position score of the three capsules was reported in the analysis. The marker capsule assessment was only included in the 20 participants included in the study by Mark et al [10].

### Intrasubject reproducibility sub-study

The physiological variation in the MRI measurements was investigated in 40 healthy volunteers (HV) using the pooled baseline data obtained in two cross-over clinical trials, each including 20 HVs, where the subjects were scanned on two baseline days before any interventions were initiated [10, 11]. The subjects were investigated with MRI at fasting and 0, 15, and 45 min after a 285-kcal meal consisting of three granola bars and 400 ml of tap water. MRI measurements included gastric half-emptying time, small bowel motility index, whole gut transit capsules (only performed in one of the trials), and volume measures of the stomach, small bowel, and colon.

### Examination duration sub-study

The influence of the number of scan time points on the calculation of gastric half-emptying time was investigated in 46 subjects with diabetes and 40 HV. The numerical values of the study have previously been reported [12]. The originally obtained dataset calculated gastric half-emptying time using gastric content volumes obtained at timepoints 0 min, 15 min, 75 min, 90 min, and 105 min after a 300 kcal and 200 ml protein shake (Nutridrik, Nutricia A/S, Lillerød, DK) mixed with 200 ml of tap water. The accuracy of the gastric volume estimation was investigated using the 86 MRI examinations, where the gastric half-emptying time was estimated using only the first three (0, 15, and 75 min) postprandial scan time points.

### Statistical analysis

Intrasubject physiological variation between baseline MRI scans acquired at two separate time points was quantified using the intraclass correlation coefficient (ICC), reported with 95% confidence intervals to assess measurement reliability. Agreement between repeated measures was further evaluated using Bland-Altman analysis, which provided limits of agreement to identify systematic bias and variability. The coefficient of variation was calculated to express relative dispersion across measurements. To compare gastric half-emptying time estimates derived from five scan time points versus a reduced protocol using three time points, the concordance correlation coefficient (CCC) was used, combining measures of both precision and accuracy to assess agreement between methods. All statistical analyses were performed using Stata (version MP17; StataCorp LP).

## Results

### Intrasubject reproducibility sub-study

**Fig. 1:**
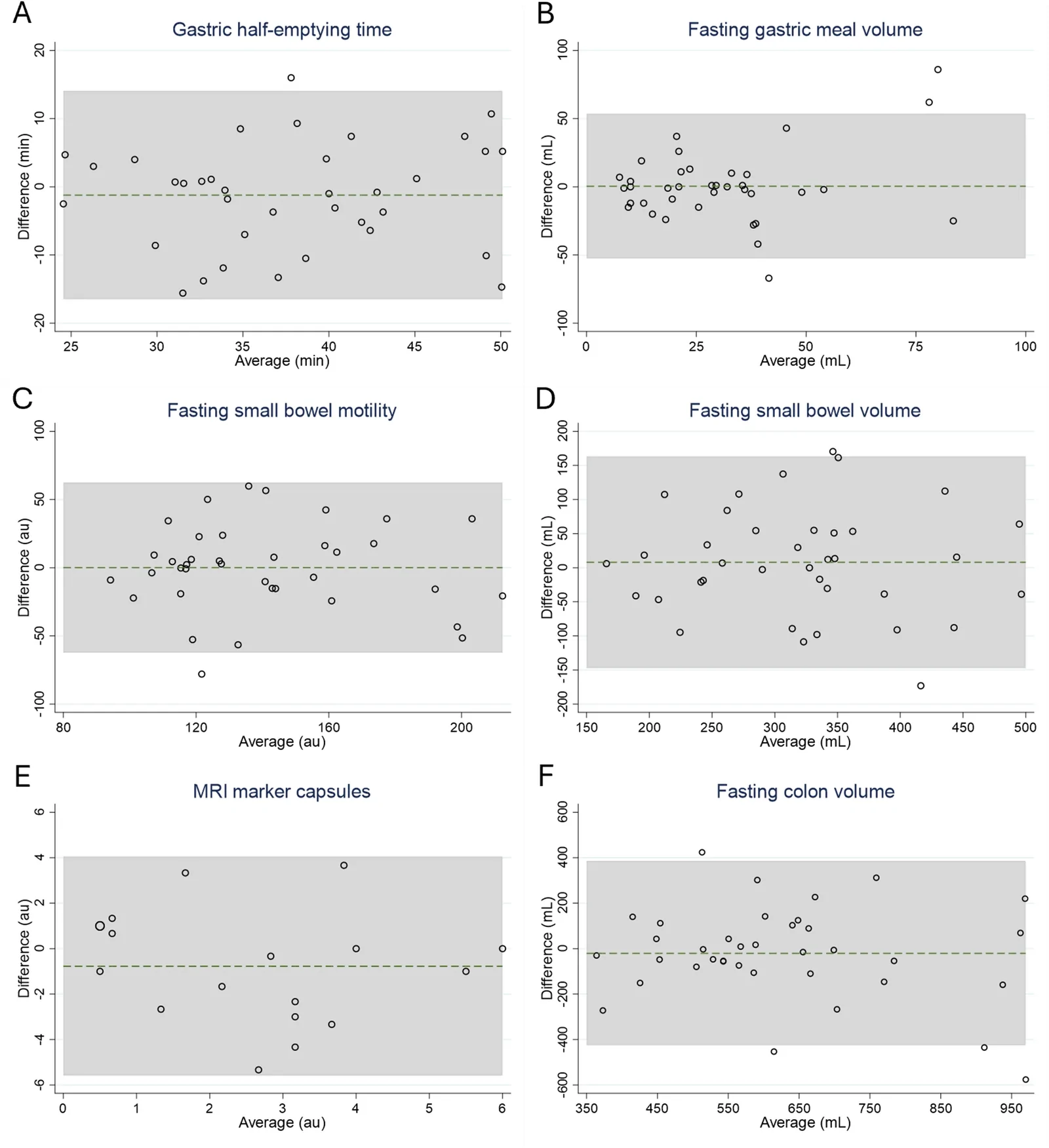
Bland-Altman plots of the physiological variation in repeated MRI measurements in 40 healthy volunteers (only 20 healthy volunteers for the MRI marker capsules). **A** Gastric half-emptying time, **B** Fasting gastric content volume, **C** fasting small bowel motility index, **D** fasting small bowel volume, **E** mean score of three MRI marker capsules representing whole gut transit, **F** fasting total colon volume

The intrasubject physiological and methodological variation in the MRI measurement were analyzed regarding gastric measurements, small bowel measurements, colonic measurements and whole gut transit times. MRI examinations were performed in 40 healthy subjects (10 females, mean age 26.1 ± 10.4 years, mean BMI 24.0 ± 3.3 kg/m2). The MRI examinations were repeated after a mean of 10.5 weeks (with a minimum of five weeks and a maximum of 24 weeks). The test-retest statistics for the repeated MRI measurements are shown in Table 2 for the fasting and the first postprandial scans directly after the meal. Bland-Altman plots for fasting values are shown in Fig. 1.

Descriptive statistics for the remaining measurements (scans performed at 15 and 45 min after the meal and for the colonic segments) are shown in the supplementary material Table S2.

#### Gastric measurements

The gastric half-emptying time demonstrated moderate reproducibility, with an ICC of 0.53 [0.31–0.74]. Gastric content volumes, however, showed poor reproducibility overall, with the highest ICC observed during the fasting scan (0.34 [0.13–0.64]). One subject showed unexplainably slow emptying of the stomach at the first visit (4.8*SD from the mean at the 45-minute scan), so when leaving that subject out of the analysis, the ICC value of the gastric content volume increased to 0.65 [0.40–0.80] at the 45-minute scan.

#### Small bowel measurements

Small bowel motility index was most reproducible during the fasting scan, with an ICC of 0.66 [0.45–0.82], and the lowest reproducibility at the 0-minute postprandial scan, with an ICC of 0.22 [0.05–0.61]. Small bowel volumes at fasting had moderate reproducibility, with an ICC of 0.61 [0.41–0.78], while the reproducibility increased postprandially showing an ICC of 0.80 [0.65–0.89]. Remaining measurements can be seen in supplementary material Table S2.

#### Colon measurements

All colonic measures were performed in a fasting state. The ICC for total colonic volume showed moderate reproducibility with a value of 0.47 [0.23–0.72]. Looking at segmental measurements, the ICC for the ascending colon was 0.48 [0.24–0.72], for the transverse colon 0.56 [0.34–0.75] and for the descending colon 0.35 [0.13–0.66]. The sigmoid colon volume showed the highest reproducibility of all the colon segments with an ICC of 0.59 [0.37–0.78].

#### Whole gut transit time

The whole gut transit capsule score showed poor reproducibility, with an ICC of 0.27 [0.04–0.76].

**Table 2:** Overview of day-to-day reproducibility on MRI-assessed endpoints in fasting and following a standardized meal in 40 healthy subjects

Measurement	*N*	Visit 1, mean ± SD	Visit 2, mean ± SD	Bland-Altman bias and LoA	CoV (intra-subject)	CoV (inter-subject)	ICC, mean [95% CI]
*Gastric content volume (ml)* *- Fasting*	37	31 ± 26	30 ± 20	0 (-53 to 53)	43	77	0.34 [0.13–0.64]
*Gastric content volume* (ml) - *Postprandial*	37	409 ± 58	387 ± 58	23 (-117 to 164)	10	15	0.20 [0.04–0.62]
*Gastric half-emptying time (min)*	36	37 ± 8	38 ± 8	-1 (-17 to 14)	12	21	0.53 [0.31–0.74]
*Small bowel motility index* (a.u.) *- Fasting*	37	142 ± 35	145 ± 41	0 (-62 to 62)	12	27	0.66 [0.45–0.82]
*Small bowel motility index* (a.u.) - *Postprandial*	37	192 ± 37	184 ± 38	9 (-82 to 100)	13	20	0.22 [0.05–0.61]
*Small bowel volume(ml)* *- Fasting*	37	324 ± 87	314 ± 93	8 (-147 to 163)	14	28	0.61 [0.41–0.78]
*Small bowel volume (ml)* - *Postprandial*	37	402 ± 119	399 ± 197	-21 (-159 to 117)	11	28	0.80 [0.65–0.89]
*Total colon volume (ml)*	37	611 ± 178	652 ± 222	-21 (-426 to 385)	17	32	0.47 [0.23–0.72]
Whole gut transit capsule score (a.u.)	18	2.2 ± 1.9	2.7 ± 2.3	-0.8 (-5.6 to 4.0)	82	86	0.27 [0.04–0.76]

The fasting scan is obtained after an overnight fast, and the postprandial scan is obtained directly after eating a 285-kcal granola bar meal with 400 ml of tap water. ICC, intraclass correlation coefficient; LoA, limits of agreement (1.96*standard deviation of difference), CoV, coefficient of variation; ICC, intra-class correlation coefficient, CI, confidence intervals.

## Examination duration sub-study

**Fig. 2:**
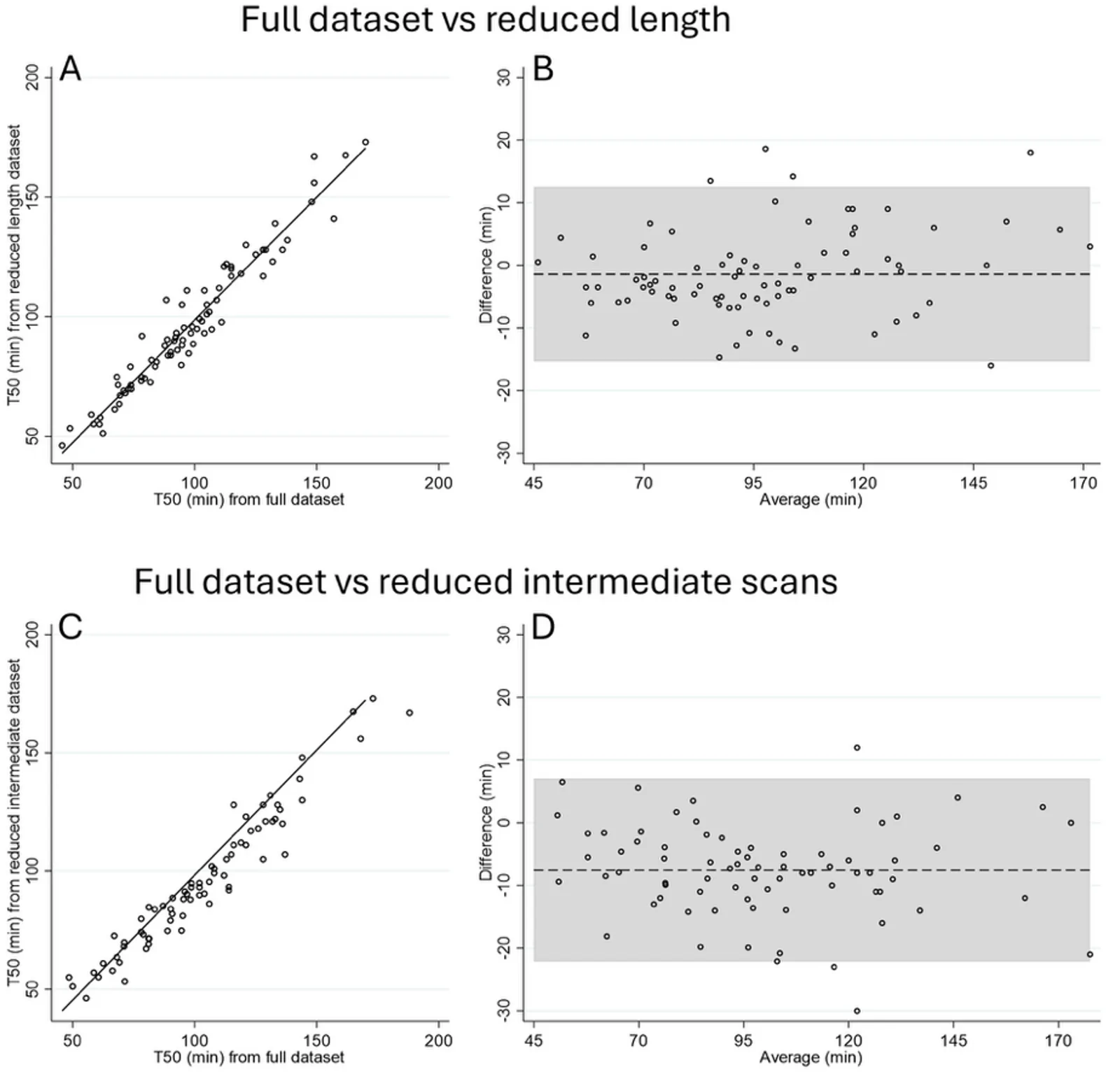
Estimations of gastric half-emptying time using reduced time spent performing the MRI examination. Top panels illustrate the impact of shortening the overall scan duration (full protocol: 0, 15, 75, 90, and 105 min; reduced protocol: 0, 15, and 75 min). **A** Scatterplot comparing half-emptying time estimates derived from the full dataset with those obtained using the reduced-duration dataset. **B** Bland–Altman plot showing the agreement between the two estimation methods. Bottom panels illustrate the effect of removing intermediate time points while maintaining total scan duration leaving room for other scan procedures (full protocol: 0, 15, 75, 90, and 105 min; reduced-intermediate protocol: 0, 15, and 105 min). **C** Scatterplot comparing estimates from the full dataset with those from the reduced-intermediate dataset. **D** Bland–Altman plot showing agreement between these two methods

The effect on gastric half-emptying estimations using fewer datapoints for calculations can be seen in Fig. 2. When leaving out scan time points performed at 90 and 105 min to reduce the length of the examination (Fig. 2A-B), the estimated gastric half-emptying time showed a bias of -1 min and 95% limits of agreement of -15 and 12 min and a concordance correlation coefficient of 0.97 when compared with the full dataset with five datapoints. When leaving out scan time points performed at 75 and 90 min (Fig. 2C-D), the estimated gastric half-emptying time showed a bias of -8 min and 95% limits of agreement of -22 and 7 min, and a concordance correlation coefficient of 0.94 between estimates of gastric half-emptying time. This indicates that the estimation of gastric half-emptying time is only changed by a few minutes when using the reduced datasets, with a tendency towards increased estimations in these cases.

**Fig. 3:**
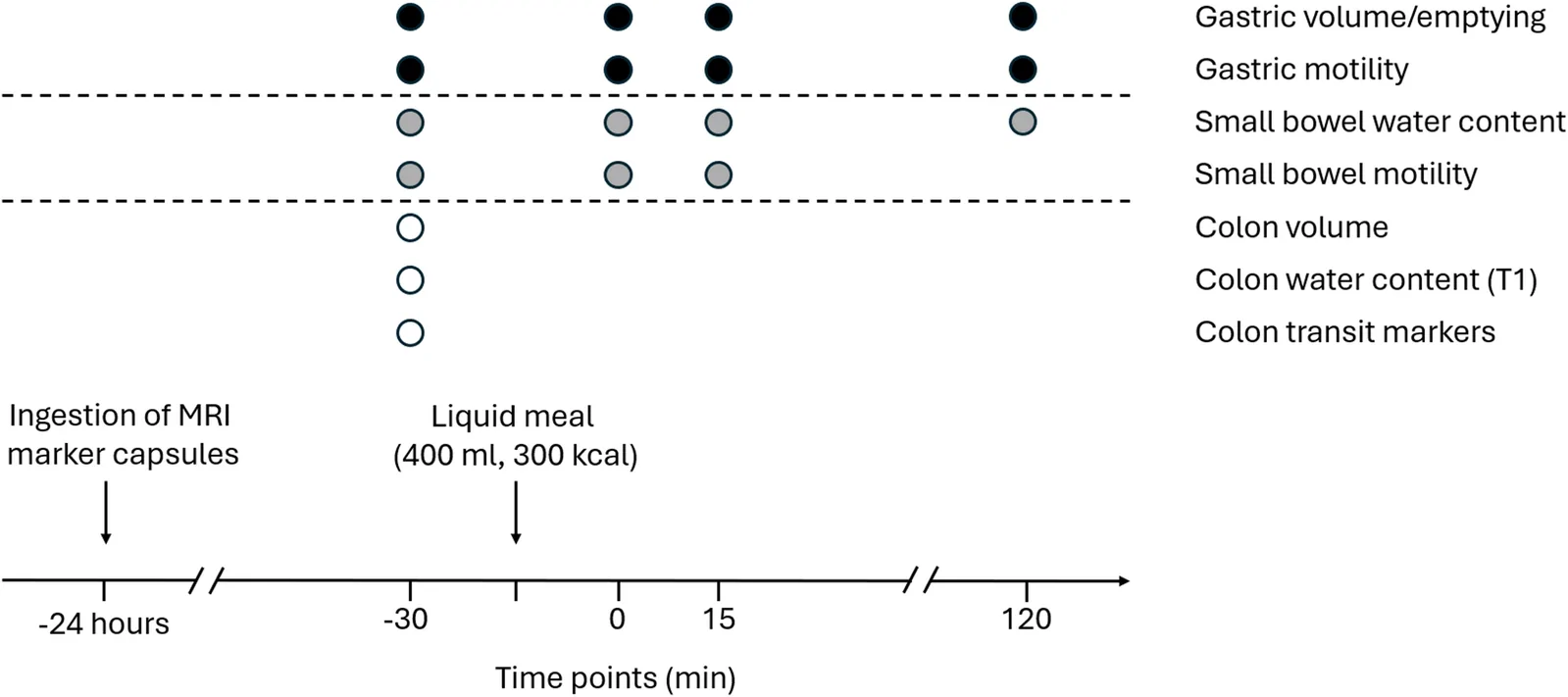
Proposal for a reduced-duration pan-alimentary MRI examination. When allowing for a break between 30 and 120 min after the test meal, the patient can leave the MRI scanner, which will reduce patient discomfort and make it possible to use the MRI scanner for other purposes in the meantime. The proposed measurement can be tailored to the specific study purpose by adding other measures or leaving out measures that are not relevant for the specific purpose

A proposal for a shortened MRI examination can be seen in Fig. 3.

### Case vignettes from Aarhus and Thisted hospitals

#### Case 1

##### Background

Male, age 69, with traumatic spinal cord injury at C2, and subsequent incomplete tetraplegia for six years. Since the injury, he spent a minimum of 1.5 h per day on the toilet due to severe constipation and arduous defecation, often rendering him with a sensation of incomplete emptying. Furthermore, he experienced appetite loss, abdominal discomfort, bloating, and pain. All different types of laxatives (stimulating, lubricating, and osmotic) had failed to alleviate symptoms. Digital stimulation/evacuation was also unsuccessful. The colonoscopy was incomplete due to a long colon and CT colonoscopy without pathological findings.

##### MRI findings

**Fig. 4:**
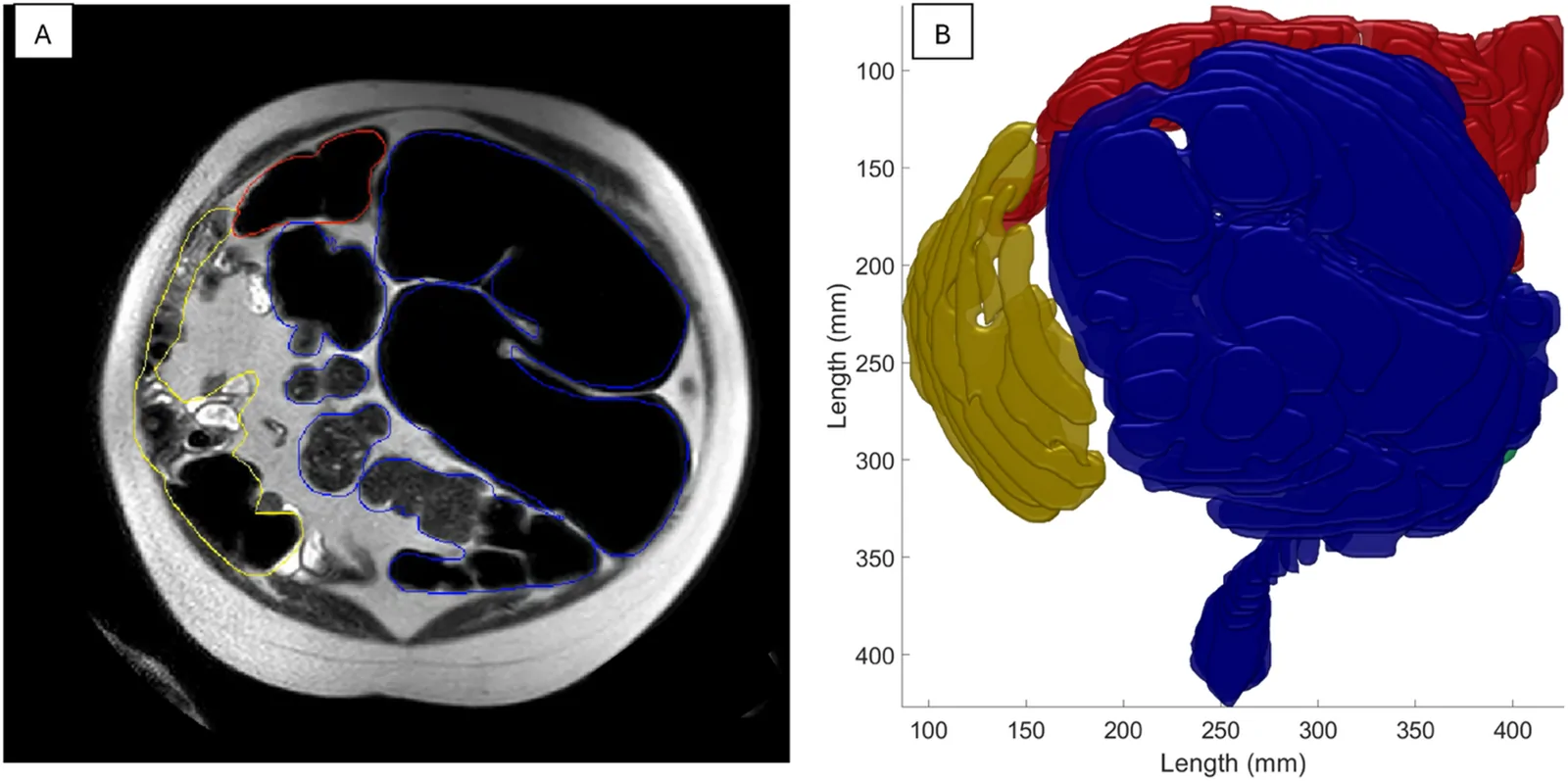
**A** Coronal T2-weighted MRI of the abdomen showing an abnormally large colon in a patient with spinal cord injury. The colon volume is segmented into the colon segments; the ascending colon is yellow, the transverse colon is red, the descending colon (not shown), and the sigmoid colon/rectum in blue. **B** 3D-model of the segmented colon volume with the same color codes as shown on the MRI

An exceptionally large colon volume of 1891 ml, see Fig. 4, where the sigmoid colon, was severely distended with feaces and gas and filled up much of the abdomen. Gastric half-emptying time was within normal range. Small bowel motility increased postprandially. The whole gut transit capsule score was measured with three contrast capsules ingested 24 h before the MRI, where all capsules were located in the proximal colon, indicating prolonged transit time. The MRI findings have been extracted from one session using this specific MRI model (Table S1.2), demonstrating that both volume, content, motility, and gastric half-emptying time can be assessed in one session.

#### Case 2

##### Background

Female, age 49, with postprandial abdominal bloating, rumbling and pain for 5–7 years. Large meals provoked symptoms. Furthermore, she experienced abdominal rumbling before defecation. Gastro- and colonoscopies were normal, as were all relevant blood- and stool tests. She fulfilled the ROME IV criteria for functional dyspepsia.

##### MRI findings

**Fig. 5:**
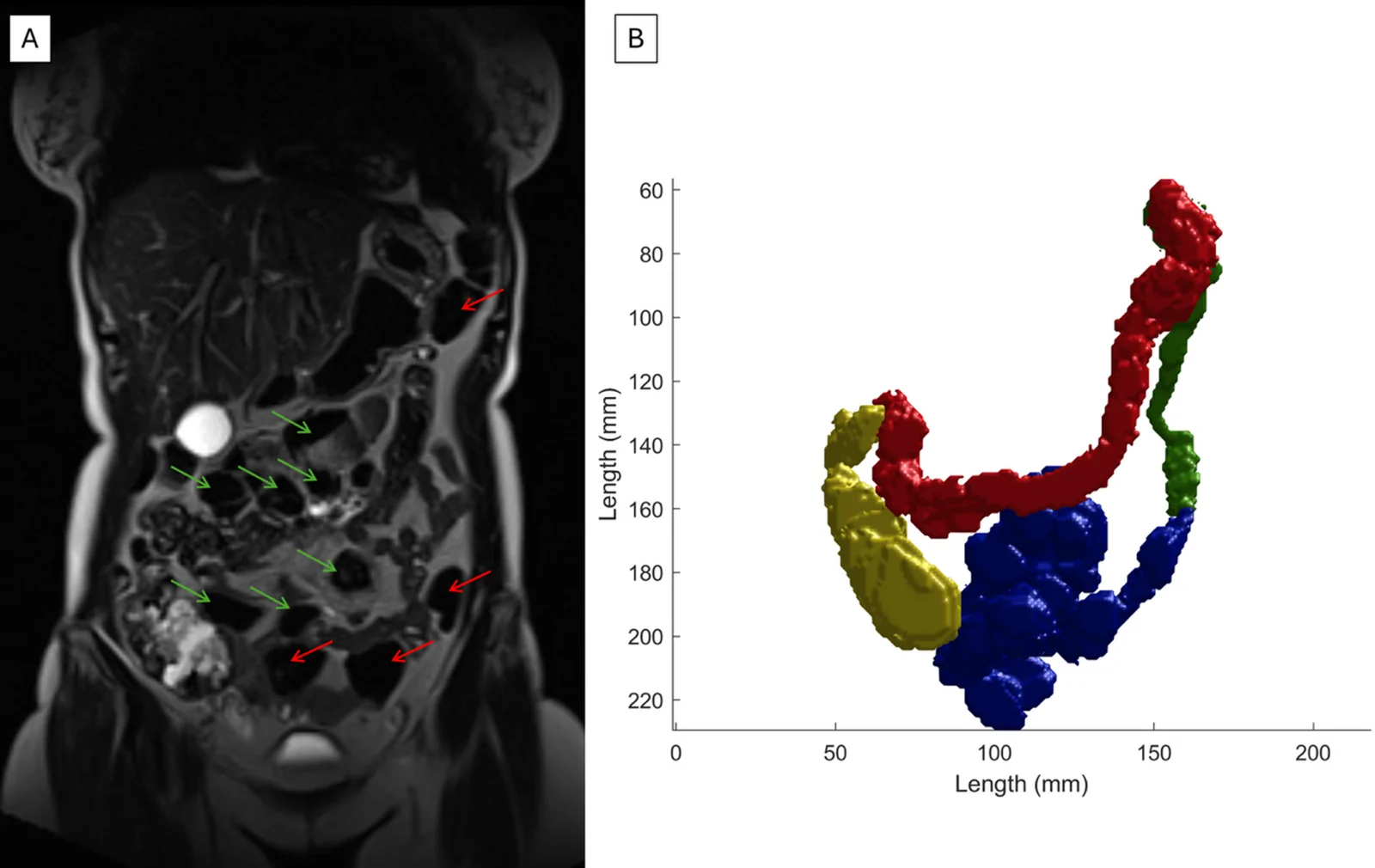
**A** Coronal T2-weighted MRI of the abdomen showing gas in the small bowel (green arrows) and in the colon (red arrows). **B** 3D-model of the segmented colon volume. The ascending colon is yellow, the transverse colon is red, the descending colon is green, and the sigmoid colon/rectum is blue

Findings included excessive amounts of air in the small intestine, see Fig. 5A, and a large total colon volume of 672 ml, as compared to the small size of the patient, see Fig. 5B. All contrast capsules were passed within 24 h, which indicates normal transit time. Normal motility was seen within the small bowel in the fasting state, and increased substantially in the postprandial phase. Gastric half-emptying time was normal. The excessive amount of air within the small intestine gave suspicion of small intestinal bacterial overgrowth.

## Discussion

This paper demonstrates that the reproducibility of MRI measurements was mostly poor to moderate, which is why physiological variations should be considered if MRI is implemented in the clinic to diagnose motility disorders. Our observations suggest that the pan-alimentary MRI protocol could be shortened so the patients do not need to stay in the scanner for extended periods. The patient cases are included to exemplify clinical feasibility of the pan-intestinal MRI examination at two different hospitals.

### Intrasubject reproducibility sub-study

#### Gastric measurements

The day-to-day intrasubject variability in fasting and postprandial gastric volumes among 40 healthy subjects was high. ICCs for repeated gastric measurements were mostly poor or moderate, with greatest repeatability seen in gastric half-emptying time. This variability likely reflects the complex physiology of gastric accommodation and emptying, which depends on multiple factors including meal form (liquid/solid), volume, caloric density, and macronutrient composition (protein, fat, fiber and carbohydrates) [18–21]. Differences in test meals across studies, as well as physiological phenomena such as gastric sieving - where liquid content of the meal leaves the stomach faster when insufficiently mixed with the solid part of the meal - may further contribute to inconsistent measurements [21, 22]. Furthermore, circadian rhythm may affect GI function and motility. Production of gastric acid increases during day, and both pancreatic enzymes and various sugar transporters likewise vary across the circadian rhythm [23]. As does motility, where increased activity in both stomach, small intestine and colon is seen to be more active during the day. In the MRI research protocols analyzed in present article, subjects were all scanned in the morning to account for circadian rhythm in both intra-and inter subject variability measurements.

In contrast to the results presented in the current study, other studies have found more reproducible results for MRI-assessed gastric emptying. Fidler et al. examined gastric responses (volume and volume changes in the fasting state and post-prandially after ingestion of a test meal) in 10 healthy volunteers on two different occasions [24]. The authors found that the coefficients of variation within subjects post-prandially were < 15%, and they concluded that fasting and postprandial gastric volumes measured with MRI were generally reproducible within subjects. Fruehauf et al., examined eight healthy volunteers and eight patients with dyspepsia on two different occasions, in fasting and post-prandial states after intake of a liquid standard meal, [25] and significant inter- and intrasubject variability in fasting gastric volumes were observed in both groups. Camilleri et al., investigated the variation in gastric emptying scintigraphy, which is more often used in the clinic, reported an intrasubject coefficient of variation for gastric half-emptying time of 24% in 47 patients, showing higher within-subject variation than found in our study [26]. The variability in gastric content volumes found in our study may partly reflect the composition of the test meal; however, gastric emptying itself is inherently variable and difficult to reproduce consistently across techniques and studies. The high variation of the volume in the fasting state seen in current paper, is consistent with a recent large study by Roelof et al., who reported that the intrasubject variability was higher than inter-subject variation [27].

Impaired gastric motility and impaired gastric emptying may be associated with a substantial symptom burden, as both accelerated and delayed emptying can be clinically relevant. Rapid emptying may be associated with suboptimal nutrient digestion, whereas delayed emptying is commonly linked to symptoms as nausea, vomiting, bloating, pain and early satiety [28, 29]. Accordingly, assessment of gastric parameters including accommodation, volume, intragastric contents and motility is relevant in the clinical evaluation of impaired gastric function. MRI can provide a safe, non-ionising and non-invasive, one-session modality that allows concurrent assessment of these parameters. However, the variability in reproducibility observed for several gastric measurements in the pooled analysis represents an important limitation, which should be considered when interpreting these findings. Nonetheless, as discussed above, given the complex nature of gastric physiology, intrasubject variability must be considered in analysis and clinical interpretation, recognizing the need for further validation and refinement of MRI protocols in the future.

#### Small bowel measurements

Disturbances in small bowel motility have been demonstrated and recognized in several conditions such as Crohn’s disease [30], chronic intestinal pseudo-obstruction [31], and small bacterial overgrowth [32]. Hence a reliable assessment of small bowel pathophysiology and motility is clinically relevant, however one needs to be wary of the limitations such as intrasubject variability, inter-subject variability and day-to-day variations as influential factors.

Intrasubject variation in repeated small bowel measurements show the ICC being poor to good and fluctuating before and after meal intake. Intriguingly, the ICC of the small bowel volume is higher and more consistent than the motility measure, also indicating moderate reliability. This aligns with previous studies on reproducibility/repeatability of bowel MRI, often demonstrating acceptable repeatability. A study by Menys et al. addressed the reproducibility of small bowel motility index using MRI in 21 healthy volunteers [14]. All subjects received bowel preparation with mannitol. Intrasubject variability for repeated baseline measurements had a coefficient of variation of 4.9%, suggesting good repeatability. The value was 12% in the current study using a meal instead of mannitol to stimulate peristalsis. De Jonge et al. investigated the response in small bowel motility to a test meal and tested the reproducibility of the MRI measurements. The study was performed in 16 subjects prepared with mannitol before the MRI and 15 subjects unprepared [33]. The intrasubject coefficient of variation for baseline fasting measurements was 23.7% in the unprepared group, whereas it was 12% in our study. The ICC of the unprepared group ranged from 0.61 at the fasting scan, similar to our findings with an ICC of 0.66 in fasting state, to 0.23 in post-prandial state, likewise aligned with our findings of an ICC of 0.22 post-prandially. Consequently, the reproducibility in our results is corroborated by other MRI studies. Despite moderate reproducibility coherent with previous findings of other authors, one still needs to be careful interpreting results, however, as aforementioned, physiological day-to-day changes within humans is a limitation we may have to accept.

#### Colonic measurements

We observed poor to moderate ICC values regarding intrasubject variation in repeated total and segmental colonic volumes. In a previous study in 20 healthy subjects, using the same imaging and analysis approach as in the current paper, we observed a higher ICC value of 0.85 in intrasubject variability of total colon and ICC values ranging from 0.44 to 0.82 for segmental colonic volumes [34]. Wilkinson-Smith et al. investigated 12 healthy volunteers, concluding a similar high ICC value of 0.84 in total colonic value and ICC values ranging from 0.63 to 0.82 for segmental colonic volumes. Although slightly higher than the results in the current paper, their results were corroborated by our previously mentioned study [35]. Nonetheless, our colonic measurements show lower intrasubject coefficient of variation values than those found in a scintigraphy transit study performed by Deiteren et al., where intra-coefficient of variations was 30% and 26% in 16 healthy subjects using 24 h measurement and 11 healthy subjects using 48 h measurements [36].

Colonic volumes can be affected in conditions such as constipation. Interestingly, some small studies suggested colonic volumes can differ dependent on the underlying pathophysiological cause [37], where others could not reproduce such results [38]. Nevertheless, different treatments may be indicated in the separate conditions. Consequently, many of the advantages with MRI could facilitate visualization of colonic features such as volume and motility, possibly enhancing clinical decisions and help adequately target patient treatment [39].

#### Whole gut transit times

The intrasubject variation of the MRI transit marker scores was surprisingly high. We replicated the methodology used in the study by Chaddock et al.; however, we only used three capsules compared to the five capsules used in the original study [16]. If this method is to be replicated again, it must be acknowledged that adding more capsules might increase accuracy. However, if transit times are not of direct clinical relevance, capsule ingestion may be omitted from the examination. Nevertheless, the proposed shortened MRI examination would remain clinically valuable, as pan-alimentary tract imaging enables comprehensive evaluation of the GI tract in a single, non-invasive test. For instance, in inflammatory bowel disease, endoscopic remission remains reference standard; however, visualization of certain parts, especially of the small bowel, is inherently limited and not necessarily possible in one single examination [40, 41]. In addition, endoscopy may be technically challenging in the presence of e.g. strictures, and it remains a semi-invasive procedure associated with patient discomfort [40]. Therefore, despite limitations, MRI inevitably poses as an eligible supplement and/or alternative in some, however not all, aspects.

### Examination duration sub-study

To enhance the clinical applicability and increase accessibility of pan-alimentary MRI, we wished to revise the examination length in an attempt to save patient discomfort, time and scanner occupancy. Scan times were scrutinized to omit redundant examinations. We evaluated the impact of reducing scan frequency in a 130-minute MRI protocol on the estimation of gastric half-emptying time. Using only three time points, baseline (0 min), early postprandial (15 min), and a later scan (either 75–105 min), the estimated half-emptying time differed by only a few minutes compared to the complete protocol. This suggests that frequent and late scans may be unnecessary for reliable estimation. While precise quantification may be essential in research settings, clinical practice often requires only a binary classification of normal versus delayed gastric emptying.

Reducing the number of scans offers advantages such as improvement of patient comfort by allowing breaks between scans, reducing scanner time, lower costs and the possibility of examining other patients in between. In nuclear medicine, Pelletier-Galarneau et al. validated a shortened scintigraphy protocol using the Bonta criteria, demonstrating that a 2-hour protocol preserved diagnostic accuracy in 95–96% of cases [42, 43]. These findings support the feasibility of streamlining MRI examinations for gastric emptying. For small bowel motility assessments, prolonged examinations may also include redundant data. Our previous work in individuals with diabetes showed that the initial postprandial response is most informative [12]. These findings are further supported by other studies likewise demonstrating significant motility changes shortly after meal intake [9, 33]. Thus, preserving early scan points appears sufficient for evaluating small bowel motility, and allowing patients to leave the scanner between later scans may not compromise diagnostic quality. In contrast, colonic assessments often require longer examination sessions. For example, the arrival of a test meal in the cecum can take a median of 225 min in healthy individuals [16]. Therefore, scan timing should be tailored to the physiological process under investigation.

### Strengths and limitations

This paper demonstrates the feasibility of implementing a shortened pan-alimentary MRI examination across multiple hospital sites using different scanner systems, supporting its potential use beyond specialized research centers. Intrasubject variation analysis was conducted on a single scanner, however, scanning across different systems would better assess inter-scanner variability. Data from each hospital were analyzed separately rather than pooled, which helps reduce site-specific bias but limits statistical power. A notable limitation is the absence of external validation. Future multi-center studies should refine the methodology, emphasizing harmonized image acquisition and post-processing techniques to enhance reproducibility and clinical applicability.

## Conclusion

Previous studies have largely focused on healthy individuals or narrow patient groups, and considerable variability still exists in scan protocols, meal provocations, and post-processing techniques. Based on different MRI examination protocols, we propose a shortened MRI examination, better suitable for future studies, recognizing the need for simplified and standardized methods. This paper demonstrates the feasibility of using pan-alimentary MRI to assess multiple gastrointestinal parameters within a single shortened examination. Despite limitations, especially regarding high intrasubject variation in MRI measures, we believe the approach remains promising, however larger studies in healthy volunteers are essential to establish normative reference values, which can then be applied in clinical studies across various disease populations.

## Supplementary Information

Below is the link to the electronic supplementary material.

Supplementary Material 1

Supplementary Material 2

## Data Availability

All data supporting the findings of this study are available within the paper and its Supplementary Information.
