# Supplementary material for: Evaluation of pan-alimentary MRI examinations: intrasubject variability and examination duration
Source: Abdom Radiol (NY). 2026 Apr 17;51(10):4858–71. doi: 10.1007/s00261-026-05399-z (PMC13577963; doi:10.1007/s00261-026-05399-z)
Supplement: Supplementary file 1 [file 261_2026_5399_MOESM1_ESM.docx]

**Supplementary Material 2**

| ***Measurement*** | ***Visit 1*** | ***Visit 2*** | ***Bland-Altman LoA*** | ***CoV (intra-subject)*** | ***CoV (inter-subject)*** | ***ICC*** |
| --- | --- | --- | --- | --- | --- | --- |
| *Gastric content volume (ml)*  *- 15 min* | 258 ± 97 | 240 ± 82 | 20.4 (-189.4 to 230.1) | 19 | 36 | 0.28 [0.08-0.62] |
| *Gastric content volume* (ml)  - *45 min* | 163 ± 70 | 148 ± 45 | 14.8 (-136.2 to 165.8) | 16 | 38 | 0.14 [0.01-0.68] |
| *Small bowel motility* (a.u.)  *- 15 min* | 149 ± 38 | 153 ± 34 | -3.2 (-84.2 to 77.8) | 15 | 24 | 0.36 [0.15-0.64] |
| *Small bowel volume(ml)*  *- 15 min* | 327 ± 86 | 328 ± 87 | -5.4 (-153.4 to 142.5) | 11 | 27 | 0.63 [0.43-0.79] |
| *Ascending colon (ml)*  *- Fasting* | 208 ± 74 | 218 ±96 | -3 (-173 to 167) | 21 | 40 | 0.48 [0.24-0.72] |
| *Transverse colon (ml)*  *- Fasting* | 183 ± 83 | 184 ± 80 | 3 (-147 to 153) | 27 | 44 | 0.56 [0.34-0.75] |
| *Descending colon (ml)*  *- Fasting* | 82 ± 48 | 103 ± 48 | -18 (-120 to 85) | 36 | 53 | 0.35 [0.13-0.66] |
| *Sigmoid colon and rectum (ml)*  *- Fasting* | 138 ± 68 | 147 ± 67 | -3 (-122 to 117) | 23 | 47 | 0.59 [0.37-0.78] |

***Table S2: Overview of day-to-day variability on MRI-assessed endpoints in fasting and following a standardized meal in 40 healthy subjects.***

*The fasting scan is obtained after an overnight fast, and the postprandial scan is obtained directly after eating a 285 kcal granola bar meal with 400 ml of tap water.*

*Abbreviations: ICC, intraclass correlation coefficient; LoA, limits of agreement, CoV, coefficient of variation; ICC, intra-class correlation coefficient.*
