# Supplementary material for: Evaluation of pan-alimentary MRI examinations: intrasubject variability and examination duration
Source: Abdom Radiol (NY). 2026 Apr 17;51(10):4858–71. doi: 10.1007/s00261-026-05399-z (PMC13577963; doi:10.1007/s00261-026-05399-z)
Supplement: Supplementary file 2 [file 261_2026_5399_MOESM2_ESM.docx]

**Supplementary Material 1**

**Table S1.1. Overview of MRI scan parameters for the 3T General Electric model SIGNA Premier**

|  | **Colonic volume** | **Stomach volume** | **Small bowel motility and volume** | **Colon transit markers** |
| --- | --- | --- | --- | --- |
| **Sequence** | SSFSE | FIESTA | FIESTA (cine) | Dixon LAVA Flex |
| **Direction/plane** | Coronal | Axial | Coronal | Coronal |
| **TE (ms)** | 90 | 1.2 | 1.2 | 2.086 |
| **TR (ms)** | Auto-calculated | 2.8 | 2.8 | 5.26 |
| **Flip Angle (°)** | 90 | 45 | 45 | 40 |
| **Slice Thickness (mm)** | 4.0 | 5.0 | 5.0 | 4.0 |
| **Slice gap (mm)** | 0 | 0 | 0 | 0 |
| **Number of Slices** | 40 | 30 | 5 | 52 |
| **Acquisition Matrix** | 0/320/256/0 | 160/0/0/200 | 160/0/0/200 | 0/300/200/0 |
| **Reconstruction Diameter (mm)** | 500 | 420 | 420 | 500 |
| **Percent Phase Field of View** | 100 | 100 | 100 | 80 |
| **Columns** | 512 | 512 | 512 | 512 |
| **Rows** | 512 | 512 | 512 | 512 |
| **Scan Duration (s)** | 47 | 20 | 20x5 | 20 |
| **No. of Breath Holds** | 2 | 1 | 5 | 1 |

**Table S1.2. Overview of MRI scan parameters for the 1.5T Siemens scanner**

|  | **Colonic volume** | **Stomach volume** | **Small bowel motility and volume** | **Colon transit markers** |
| --- | --- | --- | --- | --- |
| **Sequence** | HASTE | TRUE FISP | TRUE FISP (cine) | VIBE DIXON |
| **Direction/plane** | Coronal | Axial | Coronal | Coronal |
| **TE (ms)** | 1.5 | 2.05 | 1.5 | 2.4 |
| **TR (ms)** | 3020 | 445.1 | 309.1 | 6.7 |
| **Flip Angle (°)** | 94 | 34 | 34 | 10 |
| **Slice Thickness (mm)** | 4 | 5 | 10 | 4 |
| **Slice gap (mm)** | 0 | 0 | 0 | 0 |
| **Number of Slices** | 40 | 30 | 5 | 44 |
| **Acquisition Matrix** | 0/256/256/0 | 256/0/0/194 | 0/256/258/0 | 0/320/258/0 |
| **Reconstruction Diameter (mm)** | 500 | 430 | 500 | 400 |
| **Percent Phase Field of View** | 100 | 75.8 | 100 | 100 |
| **Columns** | 256 | 512 | 512 | 258 |
| **Rows** | 256 | 388 | 512 | 320 |
| **Scan Duration (s)** | 2x20 | 13 | 5x20 | 17 |
| **No. of Breath Holds** | 2 | 1 | 5 | 1 |

**Table S1.3. Overview of MRI scan parameters for the 1.5T Philips scanner**

|  | **Colonic volume** | **Stomach volume** | **Small bowel motility and volume** | **Colon transit markers** |
| --- | --- | --- | --- | --- |
| **Sequence** | SSFSE | bTFE | bTFE (cine) | TFE (mDIXON) |
| **Direction/plane** | Coronal | Axial | Coronal | Coronal |
| **TE (ms)** | 90 | 2.1 | 1.7 | 4.7 |
| **TR (ms)** | 6809 | 4.2 | 3.3 | 5.6 |
| **Flip Angle (°)** | 90 | 45 | 45 | 40 |
| **Slice Thickness (mm)** | 4 | 5 | 10 | 4 |
| **Slice gap (mm)** | 0 | 0 | 0 | 0 |
| **Number of Slices** | 40 | 38 | 3x3 | 140 |
| **Acquisition Matrix** | 0/312/261/0 | 212/0/0/163 | 0/280/224/0 | 0/304/202/0 |
| **Reconstruction Diameter (mm)** | 500 | 420 | 420 | 500 |
| **Percent Phase Field of View** | 100 | 90.7 | 100 | 100 |
| **Columns** | 640 | 560 | 336 | 512 |
| **Rows** | 640 | 560 | 336 | 512 |
| **Scan Duration (s)** | 2x17 | 18 | 3x20 | 18 |
| **No. of Breath Holds** | 2 | 1 | 3 | 1 |
